# ‘Breakfast: how important is it really?’ A response

**DOI:** 10.1017/S1368980016000380

**Published:** 2016-03-15

**Authors:** Hannah J Littlecott, Gary F Moore, Laurence Moore, Ronan A Lyons, Simon Murphy

**Affiliations:** 1 Centre for the Development and Evaluation of Complex Interventions for Public Health Improvement (DECIPHer)School of Social SciencesCardiff University1–3 Museum Place, Cardiff CF10 3BD, UK Email LittlecottH@cardiff.ac.uk; 2 MRC/CSO Social and Public Health Sciences UnitUniversity of GlasgowGlasgow, UK; 3 Centre for the Development and Evaluation of Complex Interventions for Public Health Improvement (DECIPHer)Centre for Health Information, Research and EvaluationSwansea UniversitySwansea, UK

Madam

First, we would like to thank the author of a recent Letter to the Editor^(^ ^1^ ^)^ for his positive overall comments on our study^(^ ^2^ ^,^ ^3^ ^)^ and for acknowledging that the findings are written with great care. While the letter moves beyond our paper to critique the wider literature surrounding the relationship between breakfast and health outcomes, such as obesity, we respond here only to those issues raised in relation to our paper. The letter rightly highlights that association does not equal causality. In our communications with the media, we consciously resisted the use of overly strong causal language for this reason. It will never be possible to randomly assign children to eat or not eat breakfast, and track their outcomes for 12–18 months in order to firmly attribute causality to breakfast itself. In the absence of such evidence, the longitudinal observational design of our study serves as the most robust form of evidence available. Of course, there remains a possibility that unmeasured confounders, such as family functioning, and clustering with other behaviours may contribute to the observed associations. Issues such as these have been debated at length with regard to breast-feeding, which continues to be strongly recommended by the public health community as providing the healthiest start in life; a paper published in 2014 in *Social Science & Medicine* ^(^ ^4^ ^)^, for example, showed that where comparisons are made between siblings who are or are not breast-fed, estimates of effects of breast-feeding on young people’s well-being were substantially reduced.

The author suggests that ‘consumption of breakfast at home is a marker for the quality of a child’s home environment and parenting … Indeed, consumption of breakfast may be a particularly good marker in this respect, better for example than free-school-meal (FSM) entitlement … as it may reflect well at an individual level the motivation and capacity of caregivers to provide a nurturing environment for their children’^(^ ^1^ ^)^. This alternative set of hypotheses is essentially premised on an assumption that a child’s consumption of breakfast is explained by ‘good parenting’. However while on the face of it, it seems plausible that parenting might contribute to this association, is the hypothesis that this fully explains the observed associations grounded in evidence?

Within the letter, the above argument is advanced without any reference to the scientific literature. We would argue that the underlying assumption that breakfast consumption is largely determined by the extent to which parents provide a nurturing home environment does not resonate all that well with the evidence. An extant literature on child feeding practice suggests that with the best will in the world, many practices commonly adopted by well-intentioned parents to encourage children to eat well, such as pressuring and restricting, are ineffective or counterproductive^(^ ^5^ ^)^. Our earlier analyses from this same study^(^ ^6^ ^,^ ^7^ ^)^ suggest that young people’s own attitudes shape their food choices and that, even at this young age (i.e. 9–11 years), they exercise and value more agency over food choices than is often assumed. Breakfast skipping increases rapidly throughout childhood, such that some estimates indicate, for example, that almost two in five girls do not eat breakfast^(^ ^8^ ^)^. It seems unlikely that this can be fully explained by failures to provide a nurturing home environment. A recent paper in this journal, for example, showed that among a sample of adolescents in Denmark, low family functioning was strongly correlated with breakfast skipping^(^ ^9^ ^)^. However, the vast majority of young people within that survey reported good family relationships, and hence the largest absolute number of breakfast skippers came from homes with high levels of family functioning. While parenting is important^(^ ^10^ ^,^ ^11^ ^)^, it is one of many socio-ecological influences on how young people eat. We would welcome further research to attempt to replicate our findings, while including measures of family functioning, in order to quantify the extent to which the observed associations are, or are not, attenuated by these variables. But we would caution against advancing alternative explanations as more likely without grounding these in evidence.

The author is also right that findings of previous studies into the link between breakfast, cognition and educational outcomes have been equivocal^(^ ^1^ ^)^; indeed we make this point at length in the introduction to our paper^(^ ^2^ ^)^. However, there are some misrepresentations of our earlier work within this, which the author presents as support for an argument that breakfast does not impact cognition. The author rightly highlights that the trial of the Primary School Free Breakfast Initiative demonstrated no effect on episodic memory, as previously reported in this journal^(^ ^12^ ^,^ ^13^ ^)^. However, whether universal breakfast provision impacts cognition is a very different question from whether breakfast does *per se*. As reported in our previous papers, the scheme led primarily to a switch from breakfast at home to breakfast at school, with limited overall impacts on breakfast skipping except among children from poorer backgrounds. While this led to small significant improvements in the quality of children’s breakfasts, the lack of intervention effect on cognition is maybe unsurprising given that the mediating mechanism (i.e. reduction in breakfast skipping) was not widely achieved. It remains to be seen whether, if breakfast schemes such as this were to effectively reach those children not having breakfast at home, this would improve their educational performance. This is an important question which remains to be empirically tested.
